# The regulation of DNA end resection by chromatin response to DNA double strand breaks

**DOI:** 10.3389/fcell.2022.932633

**Published:** 2022-07-15

**Authors:** Bo-Ruei Chen, Barry P. Sleckman

**Affiliations:** ^1^ Division of Hematology and Oncology, Department of Medicine, University of Alabama at Birmingham, Birmingham, AL, United States; ^2^ O’Neal Comprehensive Cancer Center, University of Alabama at Birmingham, Birmingham, AL, United States

**Keywords:** DNA end resection, homologous recombination, non-homologous end joining, histone modificaitons, chromatin remodeling, 53BP1, BRCA1

## Abstract

DNA double-strand breaks (DSBs) constantly arise upon exposure to genotoxic agents and during physiological processes. The timely repair of DSBs is important for not only the completion of the cellular functions involving DSBs as intermediates, but also the maintenance of genome stability. There are two major pathways dedicated to DSB repair: homologous recombination (HR) and non-homologous end joining (NHEJ). The decision of deploying HR or NHEJ to repair DSBs largely depends on the structures of broken DNA ends. DNA ends resected to generate extensive single-strand DNA (ssDNA) overhangs are repaired by HR, while those remaining blunt or minimally processed can be repaired by NHEJ. As the generation and repair of DSB occurs within the context of chromatin, the resection of broken DNA ends is also profoundly affected by the state of chromatin flanking DSBs. Here we review how DNA end resection can be regulated by histone modifications, chromatin remodeling, and the presence of ssDNA structure through altering the accessibility to chromatin and the activity of pro- and anti-resection proteins.

## Introduction

**FIGURE 1:**
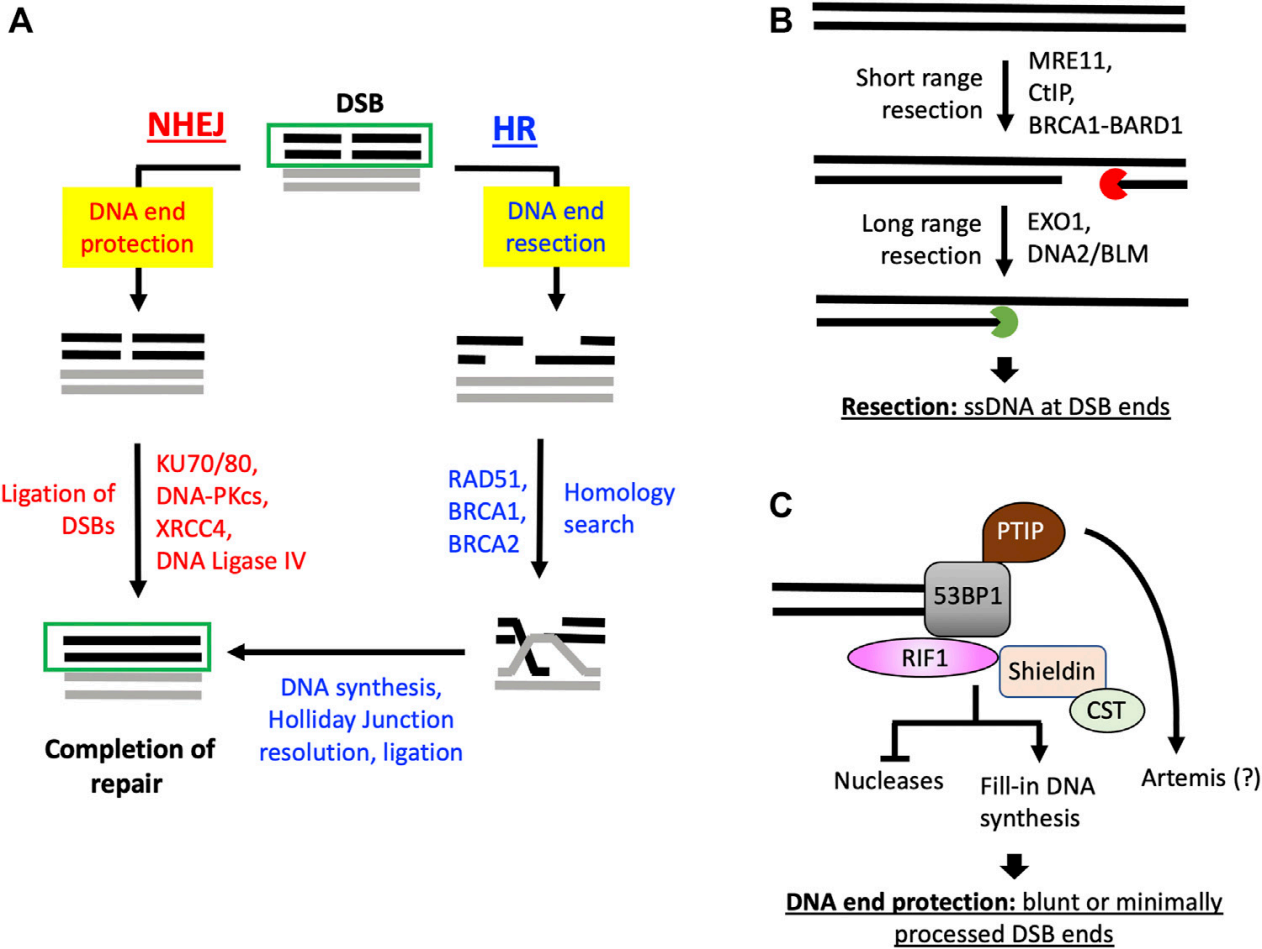
DNA resection determines DSB repair pathways choice between HR and NHEJ. **(A)** DSBs with ends protected by DNA end protection proteins can be repaired by NHEJ. DSBs may also be resected by nucleases and accessory proteins to generate ssDNA overhangs to promote HR and prevent NHEJ function at such resected DNA ends. **(B)** Distinct groups of nucleases and accessory proteins promote short-and long-range resection of DNA DSBs to generate ssDNA to initiate HR. **(C)** 53BP1, through different downstream effectors, restricts nuclease activity at DSBs and/or promotes fill-in DNA synthesis at resected DSBs to maintain blunt or limited processed DNA ends for NHEJ. The question mark (?) next to Artemis indicates that although Artemis inactivation allows for DNA resection in BRCA1-deficient cells, current evidence indicate it could indirectly affect DNA end processing (see main text).

Faithful transmission of genetic information during each cell-division cycle is key to the fitness and survival at the organismal level. This is a daunting task as DNA damage arises constantly in various forms in cells at different genomic locations and during different phases of the cell cycle. DNA double-strand breaks (DSBs) are among the most deleterious DNA lesions that can be generated spontaneously in cells during physiological processes, including DNA replication, transcription, and meiosis, or upon exposure to genotoxic agents, such as irradiation and chemotherapeutics. Regardless of the sources of DSBs, failure to repair or erroneous repair of DSBs can result in mutations, chromosomal deletion. And translocation that often lead to developmental disorders, including neurological and immunological syndromes and cancers (Jackson and Bartek, 2009; Goldstein and Kastan, 2015; Tubbs and Nussenzweig, 2017). DNA DSBs are primarily repaired by two mechanisms: non-homologous end joining (NHEJ) and homologous recombination (HR) (Prakash et al., 2015; Chang et al., 2017). HR functions in S and G_2_ phases of the cell cycle and uses a sister chromatid as the template for accurate repair (Prakash et al., 2015). NHEJ is active in the entire cell cycle although it is the primary DSB repair pathway in G_1_/G_0_ cells due to the lack of sister chromatids (Chang et al., 2017). The critical bifurcation of the DSB repair pathway choice is the processing of DNA ends, also termed DNA end resection (Cejka and Symington, 2021; Gnugge and Symington, 2021) (Figure 1A). DNA DSBs repaired by HR are first resected to generate single-strand DNA (ssDNA) overhangs that are quickly bound by ssDNA-binding complex replication protein A (RPA), which is composed of RPA70, RPA32 and RPA14, to initiate additional steps of HR (Symington and Gautier, 2011; Prakash et al., 2015; Gnugge and Symington, 2021). On the other hand, NHEJ repairs DSB by directly joining the broken DNA ends with minimal or no processing. Indeed, studies have shown that resected DSBs with long ssDNA overhangs are inhibitory to NHEJ-mediated repair (Symington and Gautier, 2011; Chang et al., 2017).

Upon DSB generation, the KU70/KU80 hetero-dimer or the MRN (MRE11/RAD50/NBS1) complex rapidly binds the broken DNA ends and initiate DSB repair by NHEJ or HR (Symington and Gautier, 2011; Chang et al., 2017; Gnugge and Symington, 2021). At DSB ends, MRN complex activates the Ataxia-Telangiectasia Mutated (ATM) kinase that phosphorylates a plethora of substrates to promote DNA damage response (DDR) and repair (Carson et al., 2003; Uziel et al., 2003; Lee and Paull, 2004). The MRE11 subunit of the MRN complex, together with CtIP, also play a critical role in initiating DNA resection at DSBs and HR-mediated repair. MRE11 has both endonuclease and exonuclease activities. Current biochemical evidence suggests that upon binding to DSBs, MRE11, with its endonuclease activity, induces a single-strand Nick some distance away from the DSB on the 5′ strand. From the single-strand Nick, the MRE11 exonuclease activity degrades DNA strand in the 3′-to-5′ direction to generate a short ssDNA overhang at the 3′-end (Wang et al., 2017; Cannavo et al., 2019) (Figure 1B). Upon the initiation of resection by MRE11-CtIP, exonucleases EXO1 and DNA2, aided by the BLM helicase, extend the length of ssDNA overhangs by promoting long-range resection in the 5′-to-3′ direction (Nimonkar et al., 2011) (Figure 1B). Although MRE11, CtIP, EXO1, DNA2 and BLM consist of the major force driving DNA resection in eukaryotic cells, additional critical factors have also been identified. For example, BRCA1, with its obligate interactor BARD1, can complex with CtIP to form one of the BRCA1-containing complexes and promote resection of DSBs (Yu and Chen, 2004; Yun and Hiom, 2009).

While resection of DNA ends paves the way for HR, long ssDNA overhangs at DSBs block repair by NHEJ. To safeguard the DSBs to be repaired by NHEJ, there are also many proteins dedicated for the protection of DNA ends from aberrant nucleolytic activity. Among the most extensively studied DNA end protection proteins is 53BP1. The function of 53BP1 in restraining resection was first discovered in BRCA1-deficient cells, where loss of 53BP1 allows for DSB resection and restoration of HR (Bunting et al., 2010). The hunt for the downstream effectors of 53BP1 subsequently led to the identification of PTIP, RIF1 and the shieldin complex (Setiaputra and Durocher, 2019) (Figure 1C). Similar to the loss of 53BP1, loss of PTIP promotes resection and HR in BRCA1-deficient cells (Callen et al., 2013). Although it remains largely unknown how PITP regulates DNA end processing, a later study indicated PTIP negatively regulates DNA resection and HR in BRCA1-deficient cells through the endonuclease Artemis (Wang et al., 2014). A surprising finding in this study indicated the loss of Artemis nuclease activity is sufficient for promoting DNA resection in BRCA1-deficient cells. While remaining to be investigated, it was speculated that Artemis may utilize its nuclease activity to trim DNA ends in a manner that promotes NHEJ and subsequently retard DNA end resection and HR indirectly (Wang et al., 2014) (Figure 1C). Numerous studies, on the other hand, have significantly advanced the mechanistic insight of the 53BP1-RIF1 pathway by the discovery of the shieldin/SHLD complex, composed of REV7, SHLD1, SHLD2 and SHLD3 (Dev et al., 2018; Findlay et al., 2018; Gao et al., 2018; Gupta et al., 2018; Mirman et al., 2018; Noordermeer et al., 2018). The proposed mechanisms of the 53BP1-RIF1-shieldin complex in DNA end protection include retarding the access of nucleolytic activity to DNA end and promoting fill-in synthesis of ssDNA regions at DSBs (Mirman et al., 2018; Noordermeer et al., 2018; Setiaputra and Durocher, 2019). The latter is supported by the interaction between the shieldin complex and the CST complex, which brings DNA polymerase α to resected DNA ends to promote DNA synthesis and restore the double-strand DNA structure (Mirman et al., 2018) (Figure 1C). It is to be noted that the two mechanisms are not mutually exclusive. In 53BP1-deficient cells, extensive DNA end resection occurs despite DNA polymerase α-dependent DNA synthesis activity is readily detectable at DSBs, indicating the fill-in DNA synthesis per se is not sufficient for preventing DNA resection (Paiano et al., 2021).

While a lot of the current mechanistic understandings of DNA end processing come from sophisticated *in vitro* studies using purified proteins on DNA or oligonucleotide substrates, the complexity of these processes is only greater in cells where DNA is wrapped around histone octamers to form nucleosomes and other higher order chromatin structure. Indeed, DSBs promote nucleosome remodeling and various histone post-translational modifications (PTMs) that alter the chromatin accessibility and generate docking sites for specific DDR or repair factors to appropriately regulate the integrity of DNA ends and subsequent choice of DSB repair pathways (Wilson and Durocher, 2017; Karl et al., 2021). Here, we review how DSB-dependent and-independent histone PTMs, chromatin remodeling and ssDNA structures regulate the access to chromatin flanking DSBs and activities of pro- and anti-resection proteins to enforce temporal and spatial control of the DNA end resection.

## Phosphorylation of histone H2AX: The foundation of DDR signaling and DSB repair focus assembly

**FIGURE 2:**
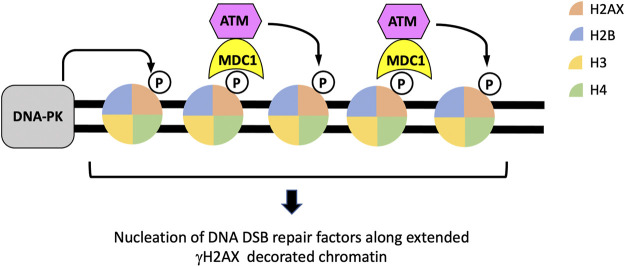
The establishment and expansion of γH2AX along chromatin regions flanking DSBs. Both DNA-PK and ATM are activated in response to DSBs and can phosphorylate histone H2AX to form γH2AX in chromatin flanking DSBs. The binding of the DNA damage response protein MDC1 and its subsequent recruitment of additional ATM proteins form a feedforward loop that extend the region of γH2AX-decorated chromatin, establishing a platform for the assembly of other repair factors.

**FIGURE 3:**
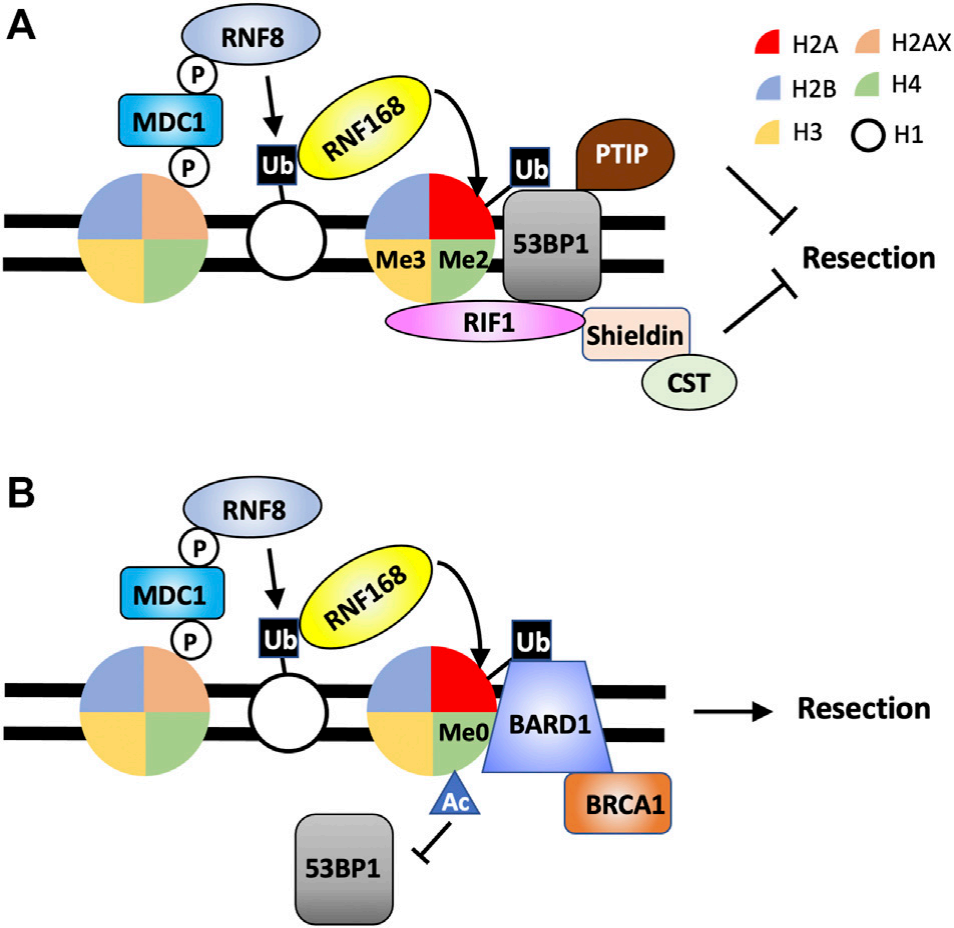
Combinations of histone PTMs determine DNA end processing by the recruitment of 53BP1-RIF1-Shieldin or BRCA1-BARD1 to DSBs. **(A)** 53BP1-RIF1-Shieldin is recruited to DSBs by H2AK15ub, H4K20me2 and H3K4me3. **(B)** BRCA1-BARD1 localizes to DSBS through the binding to H2AK15ub and H4K20me0. While H4K16Ac is not directly involved in promoting BRCA1-BARD1 recruitment to DSBs, it prevents the association of 53BP1 at damaged chromatin.

In response to DNA DSB formation, perhaps the very first histone modification event is the phosphorylation of the histone H2A variant H2AX at serine 139 to form γH2AX by the activities of the phosphatidylinositol 3-kinase-related kinases ATM, DNA-dependent protein kinase (DNA-PK), and to some extent, Ataxia telangiectasia and Rad3 related (ATR) (Rogakou et al., 1998; Rogakou et al., 1999; Blackford et al., 2017). The formation of γH2AX is a rapid process, appearing with minutes after the generation of DSBs, and can occur over a long distance, several mega bases, from the breakage site (Rogakou et al., 1999; Bonner et al., 2008; Savic et al., 2009). For this reason, the formation and resolution of γH2AX is often used as a surrogate marker for DSB generation and repair. In addition to signaling the presence of DNA breakage in cells, γH2AX serves as a platform that supports the recruitment of proteins that are important for DDR and repair. In this regard, MDC1, through its carboxyl-terminal tandem BRCA1 carboxyl-terminus (BRCT) domains, recognizes and associates with γH2AX in the chromatin regions flanking DSBs (Lee et al., 2005; Stucki et al., 2005; Savic et al., 2009). Chromatin-bound MDC1 interacts with ATM and in turn brings more ATM to damaged chromatin regions to continuously promotes the phosphorylation of H2AX and expand γH2AX-decorated chromatin around DSBs (Stewart et al., 2003; Stucki et al., 2005; Lou et al., 2006; Savic et al., 2009) (Figure 2). γH2AX also plays an important role in the nucleation of other histone-modifying enzymes and DSB repair proteins, such as RNF8, RNF168, 53BP1 and BRCA1. These factors, together with γH2AX, form distinct nuclear foci at DNA DSBs that can be visualized by immunofluorescence detection (Lukas et al., 2011) (Figures 2, 3). It is important to note that although γH2AX is required to sustain DSB-associated nuclear foci of many DSB repair factors, the initial recruitment of at least some of these proteins, such as 53BP1 and BRCA1, to damaged chromatin can occur independently of γH2AX (Celeste et al., 2003).

The role of H2AX in DNA end protection was revealed by studies demonstrating resected broken DNA ends and accumulation of ssDNA or RPA at DSBs in different phases of the cell cycle (Helmink et al., 2011; Yamane et al., 2013; Dorsett et al., 2014; Tubbs et al., 2014). This function of H2AX depends largely on the phosphorylation of serine 139 as H2AX^S139A^ mutant fail to restore DNA protection (Helmink et al., 2011). The dynamics of phosphorylation and dephosphorylation of H2AX serine 139, in addition to γH2AX formation, appears to be important for DDR. Cells expressing the phospho-mimicking H2AX^S139E^ mutant failed to form NBS1 irradiation-induced foci (IRIF), similar to cells expressing phosphor-blocking mutant H2AX^S139A^ (Celeste et al., 2003). It is worth noting that the phosphorylation state of H2AX tyrosine 142 affects the ability of γH2AX to recruit DDR and repair factors as H2AX phosphorylated at both serine 139 and tyrosine 142 cannot support the formation of IRIF of MDC1, NBS1 and MRE11 (Cook et al., 2009).

## RNF8 and RNF168-mediated ubiquitination

Ubiquitination of proteins occurs through the concerted actions of E1 activating enzymes, E2 conjugating enzymes and E3 ligases to add a 76-amino acid ubiquitin polypeptide to the lysine residues of the targeted proteins (Welchman et al., 2005). Similar to γH2AX, histone ubiquitination occurs quickly in response to DSB formation and spreads across a long distance (a few megabases) from the breakage sites (Clouaire et al., 2018). RNF8 and RNF168 are monomeric E3 ubiquitin ligases that rapidly localize to DSBs to promote histone ubiquitination leading to DDR and DSB repair (Mailand et al., 2007; Doil et al., 2009). The roles of RNF8 and RNF168 in DSB repair were first revealed by their critical requirement of IRIF of DSB repair factors 53BP1 and BRCA1 (Figure 2). Cells depleted of or deficient in RNF8 or RNF168 fail to form 53BP1 or BRCA1 IRIF (Huen et al., 2007; Mailand et al., 2007; Doil et al., 2009; Stewart et al., 2009). RNF8 is recruited to DSBs through its forkhead-associated (FHA) domain, which binds to phosphorylated MDC1 (Huen et al., 2007; Mailand et al., 2007). Although RNF8 was initially thought to promote H2A ubiquitination, biochemical and structural analyses indicate that RNF8 is inactive to H2AX or H2AX in the context of a nucleosome (Mattiroli et al., 2012). A later study showed that RNF8, both *in vitro* and *in vivo*, preferentially targets the linker histone H1 for K63-linked ubiquitination (Huen et al., 2007; Mailand et al., 2007; Thorslund et al., 2015). Ubiquitination of histone H1 could potentially serve two purposes for DSB repair. Ubiquitinated histone H1 appears to associate with chromatin more loosely compared to unmodified form, likely making chromatin more accessible to repair factors or chromatin remodelers (Thorslund et al., 2015). The K63 ubiquitin chains on modified histone H1 also serve as a recruiting platform for RNF168, which has a ubiquitin-dependent DSB recruitment module 1 (UDM1) in its amino terminus that binds strongly with ubiquitinated histone H1 (Thorslund et al., 2015). This observation is in accord with that RNF168 IRIF formation depends on the E3 enzyme activity of RNF8 (Doil et al., 2009; Stewart et al., 2009). In addition to RNF8-dependent ubiquitination, neddylation of histone H4 by UBE2M and RNF111 has been shown to support RFN168 recruitment to damaged chromatin and is particularly important in cells lacking RNF8 (Ma et al., 2013).

Once recruited to DSBs, RNF168 catalyzes ubiquitination of H2A (and H2AX) on lysines 13 and 15, although a study indicates H2AK15 to be major targeted residue by RNF168, to further amplify the ubiquitin signaling initiated by RNF8 (Doil et al., 2009; Stewart et al., 2009; Mattiroli et al., 2012; Fradet-Turcotte et al., 2013; Thorslund et al., 2015). Paradoxically, H2AK15ub is recognized by both the pro-resection proteins BRCA1-BARD1 through the BRCT domain of BARD1 and the DNA end protection protein 53BP1 *via* its ubiquitin-dependent recruitment (UDR) domain and is required for the IRIF formation of these factors (Fradet-Turcotte et al., 2013; Becker et al., 2021; Hu et al., 2021; Krais et al., 2021). Additional histone modifications therefore have been shown to create specific “histone codes” to allow for spatially and temporarily regulated localization of 53BP1 and BRCA1-BARD1 to DSBs (see below). Loss of RNF8 or RNF168 result in increased DNA end resection, consistent with the critical functions of these proteins in recruiting 53BP1 to DSBs (Chen et al., 2021a). Interestingly, depletion of RNF8 or RNF168 in 53BP1-deficient cells results in a resection phenotype less severe than that in cells lacking only 53BP1 (Chen et al., 2021a). This result suggests that loss of RNF8 or RNF168 and likely the corresponding histone ubiquitination also impair the full access of nucleolytic activities to DNA ends, although it is not clear which pro-resection factors have limited access to DNA ends in this context.

The contribution of RNF168 on DNA end processing may extend beyond histone ubiquitination. RNF168 was shown to promote 53BP1 ubiquitination in the oligomerization domain and RNF168-dependent ubiquitination of 53BP1 is important for 53BP1 IRIF formation, cellular resistance to irradiation (IR), and NHEJ (Bohgaki et al., 2013). RNF8 and RNF168 can also promote the proteosome-dependent degradation of the methyl-histone binding protein JMJD2A to expose additional histone PTMs required for 53BP1 localization to DSBs (see below) (Mallette et al., 2012).

## Histone methylation and acetylation

While γH2AX and RNF8/RNF168-dependent histone H2A ubiquitination provide a strong DSB-dependent platform for the assembly of factors regulating DNA end processing, both histone modifications are required for the retention of pro- and anti-resection proteins (e.g. BRCA1 and 53BP1, respectively) at the DSB sites. Additional mechanisms must exist to provide specificity as to the assembly of proteins at DSBs for DNA end resection or protection. In this regard, to localize to DSBs, 53BP1 also relies on its ability to recognize and bind to demethylated histone H4 at lysine 20 through its TUDOR domain (Botuyan et al., 2006) (Figure 3A). Unlike γH2AX or RNF8/RNF168-dependent H2A ubiquitination, H4K20me2 is not induced by DNA damage. Rather, existing H4K20me2 becomes exposed and is made available to 53BP1 in response to DSBs by the removal of JMJD2A and L3MBTL1. These two proteins occupy H4K20me2 through their TUDOR and MBT domains, respectively (Acs et al., 2011; Mallette et al., 2012). The binding of 53BP1 to H4K20me2 is additionally regulated by the acetylation of histone H4 at lysine 16 catalyzed by the TIP60 histone acetyl transferase (HAT) complex and is thought to disrupt the interaction between H4K20me2 and the TUDOR domain of 53BP1 (Tang et al., 2013). Interestingly, TIP60-depleted cells, similar to BRCA1-deficient cells, are defective in HR and sensitive to PARP inhibitor in a 53BP1-dependent manner, suggesting the ability of 53BP1 to block DNA resection is also regulated by histone acetylation (Tang et al., 2013).

In contrast to 53BP1, BRCA1 binding to damaged chromatin is inhibited by the presence of H4K20me2 and depends on H4 with unmethylated lysine 20 (H4K20me0) (Nakamura et al., 2019) (Figure 3B). Majority of histone H4 in cells are methylated at lysine 20, and only in S phase, newly synthesized, unmodified H4 are incorporated in nucleosomes in the nascent chromatin after DNA replication (Rice et al., 2002; Pesavento et al., 2008; Saredi et al., 2016). Therefore, H4K20me0 serves as a good temporal regulator for DSB events that normally take place in S phase, such as HR. For example, the protein complex TONSL-MMS22 recognizes H4K20me0 in nascent chromatin to promote HR in S phase (Saredi et al., 2016). Similarly, the pro-resection complex BRCA1-BARD1 also specifically recognizes H4K20me in post-replicative, nascent chromatin in S phase through the ankyrin repeat domain (ARD) of BARD1. Mutations in the ARD of BARD1 impair the recruitment of BRCA1-BARD1 complex to DSBs in S phase, DNA end resection and the efficiency of HR (Nakamura et al., 2019; Becker et al., 2021).

Therefore, both 53BP1 and BARD1 of the BRCA1-BARD1 complex are bivalent nucleosome readers. Both proteins recognize and bind H2AK15ub with 53BP1 using its UDR domain and BARD1 *via* its BRCT domain (Fradet-Turcotte et al., 2013; Becker et al., 2021; Hu et al., 2021). However, the localization of 53BP1 and BARD1 to DSBs also requires simultaneous binding to distinctly modified histone H4, with 53BP1 TUDOR domain targeting H4K20me2 and BARD1 ARD binding to H4K20me0 (Botuyan et al., 2006; Nakamura et al., 2019; Becker et al., 2021). In response to DSBs, specific combinations of histone PTMs fine tune the decisions on DNA end processing and subsequent DSB repair choice. To make the already complicated histone codes more complex, a new study uncovered that, in addition to 53BP1, its downstream effector RIF1 also can recognize modified histone, H3K4me3 promoted by SETD1A/BOD1L1 methyltransferase complex (Bayley et al., 2022) (Figure 3A). Loss of SETD1A/BOD1L1-dependent H3K3me3 impairs the accumulation of RIF1 at DSBs and promotes DNA resection while not affecting the recruitment of 53BP1 to DSBs (Bayley et al., 2022). Thus, the 53BP1-dependent DNA end protection pathway is enforced by a combination of at least three independent histone modifications.

## Poly ADP-ribosylation

In response DSBs and many other types of DNA damage, poly ADP-ribose polymerases (PARPs) use nicotinamide adenine dinucleotide (NAD+) to catalyze covalent modification of many DNA repair factors with single or multiple ADP-ribose units at glutamine, asparagine or lysine residues to facilitate the repair of DNA lesions (Ray Chaudhuri and Nussenzweig, 2017). Core histones and the linker histone H1 are among the targets of PARPs and DNA damage-dependent histone PARylation has been shown to promote transient histone removal from and relaxation of damage chromatin, presumably increasing chromatin accessibility to DNA repair factors (Poirier et al., 1982; Messner et al., 2010; Strickfaden et al., 2016). It is to be noted that PARPs are also important regulators of other nuclear functions such as transcription. For example, PARP1 has been shown to enrich at the promoters of many RNA polymerase II-transcribed genes and is responsible for excluding linker histone H1 in these regions to maintain an open chromatin environment to support active transcription (Krishnakumar et al., 2008).

Among the large PARP family members, PARP1 is known for its roles in the diverse DNA damage repair pathways, including single strand break (SSB) repair, base-excision repair (BER), nucleotide excision repair (NER) and DSB repair (Schreiber et al., 2006; Ray Chaudhuri and Nussenzweig, 2017). PARP1 and other PARP family member have been PARP1 has a DNA binding domain composed of 3 zinc finger motifs in its amino terminus and can recognize and bind DNA breaks (Ali et al., 2012; Langelier et al., 2012; Sukhanova et al., 2016). Early evidence suggesting that PARP1 may be involved in DSB repair came from the observations that PARP inhibitor-treated cells exhibited increased DNA damage-induced sister chromatid exchange (SCE) while increased expression of PARP1 decreased SCE, a process that depend on HR (Oikawa et al., 1980; Hori, 1981; Meyer et al., 2000). While it remains not entirely clear how mechanistically loss of PARP1 may promote DSB repair through HR, recent studies suggest two potential mechanisms (Hu et al., 2014; Caron et al., 2019). First, in response to DSBs, the BRCA1 was shown to undergo PARP1-mediated PARylation to promotes its association with the RAP80 complex and BRCA1 mutations that prevent PARylation decrease BRCA1-RAP80 interaction (Hu et al., 2014). RAP80, together with ABRAXAS, BRCC36, BRCC45 and MERIT40, can restrict the HR promoting function of BRCA1 to thwart hyper-recombination. Interestingly, loss of RAP80 (or its associating factors) and mutations that impair BRCA1 PARylation, and therefore BRCA1-RAP80 interaction, both stimulate HR as shown in increased SCE and elevated short and long track gene conversion using fluorescent reporter constructs, respectively (Hu et al., 2011; Hu et al., 2014). More recently, it was shown that PARP1 can restrict HR through regulating DNA end resection (Caron et al., 2019). PARP1 seems to limit aberrant DNA resection by functioning as a physical barrier to nucleases EXO1 and DNA2 as both WT and a catalytically inactive form of PARP1, when bound to DNA ends, prevent EXO1- and DNA2-mediated resection *in vitro* (Caron et al., 2019). The same study also demonstrated that the DNA end protection proteins 53BP1 and RIF1 do not form DSB-induced foci efficiently even in G_1_-phase cells, in which DNA resection is normally restricted, indicating that PAPR1 could influence DNA end processing through multiple mechanisms (Caron et al., 2019). It is to be determined how PARP1 regulate the nucleation of 53BP1 and its downstream effectors at DNA DSBs.

Despite existing evidence indicating a role for PARP1 in limiting DNA end resection and HR, PARP1 has also been shown to interact with MRE11 and promote the localization of the MRN complex, which is critical for the initiation of DNA resection, to laser-induced DSBs (Haince et al., 2008). Along the line, independent studies demonstrating that PARP1 mediates MRE11-dependent nucleolytic degradation of stall replication forks, indicating that PARP1 can promote resection in certain contexts (Bryant et al., 2009; Ding et al., 2016). Therefore, the roles of PARP1 on DNA resection appear highly complex and context dependent. In addition, although PARP-mediated chromatin relaxation is known to have significant impact on transcription, whether it also affect DNA end processing remains to be determined (Ray Chaudhuri and Nussenzweig, 2017).

## Chromatin remodeling

Nucleosomes have critical functions in genome organization and numerous biological processes occurring in the nucleus. It is conceivable that nucleosome occupancy and stability in chromatin affect DNA resection. Indeed, *in vitro* studies have demonstrated nucleosomes also negatively impact the ability of several nucleases to process DNA ends, as compared to naked DNA (Adkins et al., 2013). Specifically, the presence of nucleosomes prevents DNA2/BLM from resecting DNA wrapping around histone octamers unless nucleosome-free DNA ends are present. In addition, nucleosomes exert much stronger block on EXO1 mediated-DNA resection regardless the presence of free DNA ends, suggesting different resection machineries may use different pathways to overcome nucleosome block in order to processing DNA ends (Adkins et al., 2013). DNA2/BLM complex appears to rely on the helicase component BLM for generating a permissive chromatin state for DNA2 activity, and EXO1, whose activity is primarily inhibited by canonical H2A/H2B tetramer in a nucleosome, is more active when histone variant H2AZ replaces H2A in the nucleosomal octamers, likely through the activity of the SWR1 remodeler (yeast) and the p400/TIP60 complex (mammals) (Xu et al., 2012; Adkins et al., 2013). Along the same line, nucleosome clearance around DSBs has been suggested to be important for resection of DNA ends in yeast. Yeast mutants, in which nucleosome eviction is inhibited, exhibit defect in generating ssDNA around DSBs (Peritore et al., 2021). It is worth noting that in yeast, nucleosome occupancy can also be regulated by means of global histone degradation, a process depends on proteosome and INO80 chromatin remodeler (Hauer et al., 2017). As expected, loss of histones results in chromatin decompaction and increased recombination-based repair, presumably in part due to more efficient DNA end processing (Hauer et al., 2017). It is unclear whether similar mechanisms exist in mammals.

SMARCAD1 and its homolog in yeast FUN30 are ATP-dependent chromatin remodelers that are capable of repositioning, exchange of nucleosomes, and *de novo* nucleosome assembly (Awad et al., 2010; Markert et al., 2021). Yeast FUN30 was first implicated as a factor promoting DNA end resection in two genetic screens for mutants that promote DSBs repairs through inhibition of resection (Chen et al., 2012; Costelloe et al., 2012). The same and other independent studies showed that SMARCAD1 also functions to promote DNA resection at DSBs in mammalian cells (Costelloe et al., 2012; Bantele et al., 2017; Chakraborty et al., 2018). Both SMARCAD1 and FUN30 localize to DNA DSBs (Chen et al., 2012; Costelloe et al., 2012; Bantele et al., 2017; Chakraborty et al., 2018), and the ATPase activities of these proteins are critical for their resection promoting activity (Costelloe et al., 2012; Densham et al., 2016; Chakraborty et al., 2018). However, it is unclear whether the nucleosome remodeling activity of SMARCAD1/FUN30 *per se* accounts for its pro-resection function *in vivo* (Adkins et al., 2013; Peritore et al., 2021). Indeed, while it is possible that SMARCAD1 and FUN30 modify DSB-flanking nucleosomes to an accessible chromatin state for nucleases, independent studies have shown that these proteins may promote DNA resection through restricting the concentrations of 53BP1 and its yeast homolog RAD9. This notion is supported by the observations that the levels of chromatin associated RAD9 is increased at sites adjacent to DSBs in *fun30Δ* yeast mutants. In mammalian cells, SMARCAD1 repositions 53BP1 away from DSBs that are repaired by HR (Chen et al., 2012; Densham et al., 2016). Both SMARCAD1 and FUN30 have putative ubiquitin-binding elements called the coupling of ubiquitin conjugation to ER degradation (CUE) motifs (Neves-Costa et al., 2009; Awad et al., 2010; Densham et al., 2016). While a direct role of the CUE motif in FUN30 in DNA resection has not been determined, yeast cells expressing CUE-deleted or mutated FUN30 exhibit moderate defect in maintaining of gene silencing in heterochromatic regions, indicating the CUE motif is important for the full activity of FUN30 (Neves-Costa et al., 2009). The CUE motifs in SMARCAD1, on the other hand, has been shown to bind mono-ubiquitinated H2A, a modification promoted by the E3 ligase activity of chromatin-bound BRCA1-BARD1. Cells expressing CUE-mutated SMARCAD1, which exhibits diminished recruitment to DSBs, are defected in RAD51 IRIF formation and HR (Densham et al., 2016), providing a crosstalk between histone ubiquitination and chromatin remodeling in response to DNA DSBs.

## DNA end processing regulated by ssDNA

When DSBs are resected or during DNA replication where ssDNA accumulates at DNA ends and elongating replication fork, RPA binds ssDNA rapidly with high affinity (Wold and Kelly, 1988; Kim et al., 1992; Wold, 1997). For this feature, chromatin regions containing ssDNA in cells are quickly coated with RPA that can be visualized by immunofluorescence imaging or flow cytometry. Important functions of RPA include stabilization of ssDNA by preventing nucleolytic degradation of ssDNA and preventing ssDNA from annealing intra- or intermolecularly to form secondary structures that would impede the proper repair of resected DSBs (Sugiyama et al., 1998; Chen et al., 2013; Deng et al., 2014). Several *in vitro* studies have suggested that RPA can bind ssDNA as short as 20–30 bases (Wold, 1997). Therefore, it is conceivable that upon the initiation of resection by MRE11 and CtIP, the RPA bound to these short ssDNA overhangs could regulate the subsequent DNA processing activities. Indeed, RPA has been shown to constrain the polarity of DNA2 nuclease activity across species (e.g yeast, human and *xenopus*) to 5′-->3′ direction on dsDNA or DNA substrates with flaps (Cejka et al., 2010; Niu et al., 2010; Nimonkar et al., 2011; Yan et al., 2011). In yeast, RPA has also been shown to physically interact with DNA2 in biochemical assays, and RPA is required for DNA2 recruitment to nuclease-induced DSBs (Bae et al., 2003; Chen et al., 2013). RPA also enhances the nuclease activities of EXO1 and DNA2 *in vitro* although the stimulating effect is stronger on DNA2 (Nimonkar et al., 2011). Moreover, RPA, through the large subunit RPA70, interacts with the BLM and stimulates its helicase activity, which together with DNA2 and EXO1, promote long-range resection (Brosh et al., 2000; Kang et al., 2018; Soniat et al., 2019). Therefore, ssDNA-bound RPA in chromatin regions flanking DSBs can form a positive-feedback loop to promote extensive, long-range resection. Upon binding to ssDNA, RPA can be phosphorylated by ATM, ATR or DNAPK as part of the DDR and DNA damage repair process, primarily at the RPA32 subunit (Anantha et al., 2007; Marechal and Zou, 2015). Interestingly, in contrast to the aforementioned pro-resection function, RPA can also negatively regulate the resection machinery upon phosphorylation (Soniat et al., 2019). Recent studies have suggested that phosphorylated RPA32 competes with the BLM helicase for the binding to the RPA70 subunit, which initiates a negative feedback loop that inhibit further DNA resection by downregulating the activity of BLM and processivities of BLM/DNA2 and BLM/EXO1 complexes (Soniat et al., 2019).

Perhaps counterintuitively, RPA also serves as recruiting platform for proteins that antagonize DNA end resection. DNA Helicase B (HELB) was originally identified as a protein important for response and recovery to replicative stress (Guler et al., 2012). The same study also demonstrated that HELB interacts with RPA through its helicase domain (Guler et al., 2012). HELB was later identified in a proteomic screen for RPA-interacting proteins that may regulate the processing of DNA DSB ends (Tkac et al., 2016). The recruitment of HELB to DNA damage sites depends on the presence of ssDNA as cells lacking CtIP, which is required to initiate DNA resection, show reduced HELB localization to laser microirradiation-induced damage stripes. Furthermore, HELB mutant that cannot interact with RPA fails to accumulate at DNA breakage sites (Tkac et al., 2016). Importantly, cells lacking HELB protein expression exhibit increased levels of ssDNA after exposure to DSB-inducing agent neocarzinostatin (NCS). Combined depletion of EXO1 and BLM blocks ssDNA accumulation in HELB*-*deficient cells after NCS treatment (Tkac et al., 2016). Together, these studies suggest that HELB acts as a feedback inhibitor of long-range resection upon detecting and binding to ssDNA through its interaction with RPA at DSB ends.

The SHLD2 subunit of the shieldin complex, the most recently identified downstream effector of 53BP1-mediated end protection pathway also exerts its function through recognition of the ssDNA at DSBs. SHLD2 has three oligosaccharide/oligonucleotide binding (OB) folds at its carboxyl terminus that are well characterized ssDNA-binding domains (Dev et al., 2018; Findlay et al., 2018; Gao et al., 2018; Noordermeer et al., 2018). SHLD2 mutants that contain mutations in the critical tryptophan residues in the first OB fold exhibit drastically reduced affinity to ssDNA *in vitro* and partially impaired SHLD2 IRIF formation (Dev et al., 2018; Noordermeer et al., 2018). It is worth noting that in 53BP1-deficient cells, in which SHLD2 fails to localize to DSBs, forced targeting of the ssDNA binding defective SHLD2 OBA (OB fold A) mutant to DSBs by RNF8 FHA domain (binds DSB-associating γH2AX) still can not block aberrant resection. These results indicate that the ssDNA-binding ability of SHLD2 is critical for its DNA end protection activity in addition to its localization to DSB sites (Noordermeer et al., 2018). It is unclear why ssDNA binding of SHLD2 is critical for DNA end protection as the recruitment of the shieldin complex to DSBs largely depends on its ability to associate with 53BP1 and RIF1.

## Conclusion and future directions

DNA DSBs occur continuously in living organisms due to normal physiological processes. Timely restoration of these deleterious DNA lesions using the proper repair mechanism is extremely critical for the maintenance of genomic stability and cellular fitness. Adding another complexity of DSB repair in cells is that DNA is wrapped around histone octamers and together with linker histone H1 to form high order chromatin structure. Here we reviewed some highlights in the field that have significantly advanced our understanding in how chromatin changes in response to DSBs direct the processing of broken DNA ends, an event playing a major role in determining whether NHEJ or HR is employed for repair. These changes include covalent modification of core or linker histones to create binding sites for different sets of resection proteins and DNA end protection proteins. Importantly, some of these proteins, such as 53BP1 and BARD1, are bivalent histone PTM readers and therefore through recognizing specific combinations of histone PTMs provide temporal and spatial specificity as to the choice of DNA end protection or resection. Some histone PTMs and chromatin remodeling activities also regulate chromatin compaction and nucleosome occupancy in regions flanking DSBs to modulate the accessibility of nucleases or other repair factors to damaged chromatin. More importantly, ssDNA generated during the early phase of DSB repair also serves as an important regulator to either prevent extending the ssDNA tract or further promote extensive resection.

An interesting question regarding DNA end processing is whether the DNA end protection and resection pathways can function together to promote DSB repair. While 53BP1 and BRCA1-BARD1 recognize incompatible chromatin features associated with DSBs, studies have indicated that 53BP1 can colocalize with BRCA1, other pro-resection proteins and even RPA and RAD51 at the same DSBs, suggesting at least in the context of HR repair, both promotion and restriction of DNA resection is of functional consequence (Reindl et al., 2015; Reindl et al., 2017; Ochs et al., 2019; Whelan and Rothenberg, 2021). High resolution imaging analyses have shown that while 53BP1 can colocalize with pro-resection and ssDNA binding proteins on chromatin with DSBs, it mostly resides in chromatin “microdomains” distal to the broken DNA ends, seemingly setting a boundary for nucleolytic activity (Ochs et al., 2019; Whelan and Rothenberg, 2021). This concept is supported in a study showing that in the absence of 53BP1, DSB repair in S phase cells switches from RAD51-dependent HR pathway to RAD52-dependent mutagenic single-strand annealing (SSA) pathway due to hyper resection (Ochs et al., 2016). It will be of importance to further investigate that when DNA end protection and resection proteins are both engaged to promote DSB repair through canonical HR (Ochs et al., 2016), how histone PTMs are deposited along the chromatin regions flanking DSBs so these factors of opposing activities can execute concerted and accurate repair.

The cell cycle-specific deposition of H4K20me0 and H4K20me2 in chromatin, together with the sophisticated structural analyses and functional characterization of the respective binding domains in 53BP1 and BARD1, have provided important insights into the mechanisms underlying the regulation of cell cycle-dependent DSB repair pathway choice. However, it has been shown that during G_1_ phase of the cell cycle, inactivation of 53BP1 or RIF1 promotes robust BRCA1 IRIF formation. Conversely, inactivation of BRCA1 allows for more RIF1 IRIF to form in S/G_2_ phase (Escribano-Diaz et al., 2013). One possible explanation for this observation is that the loss of RIF1 creates “binding sites” at DSBs for BRCA1 and vice versa. However, this does not seem to be the case since a (human) RIF1 mutant lacking the C-terminal 197 amino acids (full length = 2,446 amino acids) was able to localize to DSBs normally but still failed to suppress BRCA1 IRIF in G_1_ phase cells (Escribano-Diaz et al., 2013). What is also intriguing is that in G_1_ phase cells, in which majority of H4K20 are methylated, BRCA1-BARD1 is able to localize to damaged chromatin in the absence of H4K20me0. In the absence of 53BP1 and/or RIF1, would H2K15ub alone be sufficient to tether BRCA1-BARD1 at DSBs or additional histone PTMs in G_1_ phase cells are required? Along the same line, G_0_-phase or quiescent cells, like cells in G_1_ phase DSBs are protected from nucleolytic resection in 53BP1-dependent manner (Chen et al., 2021b). However, inactivation of the transcriptional repressor DREAM complex results in increased expression of multiple factors that are critical for HR-mediated repair, including BRCA1 and BARD1, in quiescent cells. This leads to unrestrained resection at DSBs that depends on BRCA1 and BARD1 (Chen et al., 2021b). Moreover, this BRCA1-BARD1-dependent resection occurs in the presence of functional 53BP1-dependent protection pathway, further demonstrating that BRCA1-BARD1 is active and can target damage-chromatin even in quiescent cells (Chen et al., 2021b). Therefore, additional spatial (interchromosomal and intrachromosomal) and temporal (different cell cycle phases) regulation of nucleosome modifications and chromatin remodeling in response to DSBs remain to be elucidated to understand the increasing complexity of pro- and anti-resection activities at DSBs.
